# Trends in Head and Neck Cancer Mortality from 1999 to 2019 in Japan: An Observational Analysis

**DOI:** 10.3390/cancers15153786

**Published:** 2023-07-26

**Authors:** Tsukasa Higashionna, Keisaku Harada, Akinari Maruo, Takahiro Niimura, Elizabeth Tan, Quynh Thi Vu, Takayoshi Kawabata, Soichiro Ushio, Hirofumi Hamano, Makoto Kajizono, Yoshito Zamami, Keisuke Ishizawa, Ko Harada, Shiro Hinotsu, Mitsunobu R. Kano, Hideharu Hagiya, Toshihiro Koyama

**Affiliations:** 1 Department of Pharmaceutical Biomedicine, Graduate School of Medicine, Dentistry, and Pharmaceutical Sciences, Okayama University, Okayama 7008558, Japan; t.higashionna@cc.okayama-u.ac.jp (T.H.); halabow1971@gmail.com (K.H.); maruo-a@okayama-u.ac.jp (A.M.); 2 Department of Pharmacy, Okayama University Hospital, Okayama 7008558, Japan; kawabata-t@okayama-u.ac.jp (T.K.); s-ushio@okayama-u.ac.jp (S.U.); hamano.hirofumi@okayama-u.ac.jp (H.H.); kajizo-m@cc.okayama-u.ac.jp (M.K.); 3 Department of Pharmacy, Kitakyushu City Yahata Hospital, Fukuoka 8058534, Japan; zamami-y@okayama-u.ac.jp; 4 Department of Clinical Pharmacology and Therapeutics, Institute of Biomedical Sciences, Tokushima University Graduate School, Tokushima 7708503, Japan; niimura@tokushima-u.ac.jp (T.N.); ishizawa@tokushima-u.ac.jp (K.I.); 5 Graduate School, Centro Escolar University Manila, Manila 1008, Philippines; eytan@usc.edu.ph; 6 Department of Pharmacy, University of San Carlos, Cebu City 6000, Philippines; 7 Faculty of Pharmacy, Haiphong University of Medicine and Pharmacy, Haiphong 04254, Vietnam; vtquynh@hpmu.edu.vn; 8 Department of Medicine, Icahn School of Medicine at Mount Sinai, Mount Sinai Beth Israel, New York, NY 10029, USA; me422084@s.okayama-u.ac.jp; 9 Department of Biostatistics and Data Management, Sapporo Medical University, Hokkaido 0608556, Japan; hinotsus@sapmed.ac.jp; 10 Department of Pharmaceutical Biomedicine, Graduate School of Interdisciplinary Science and Engineering in Health Systems, Okayama University, Okayama 7008530, Japan; mitkano@s.okayama-u.ac.jp; 11 Department of General Medicine, Okayama University Graduate School of Medicine, Dentistry and Pharmaceutical Sciences, Okayama University, Okayama 7008558, Japan; hagiya@okayama-u.ac.jp

**Keywords:** head and neck cancer, mortality, joinpoint regression, trend analysis

## Abstract

**Simple Summary:**

The number of cases of head and neck cancer (HNC) and related deaths has recently increased worldwide. To the best of our knowledge, few studies have examined crude or age-adjusted HNC mortality rates in Japan. Therefore, this study aimed to determine the trends in crude and age-adjusted mortality rates for HNC per million individuals in Japan from 1999 to 2019. In Japan, the number of HNC-related deaths increased 1.48-fold. Age-adjusted mortality rates for HNC were four times higher in men than in women, and the rates for both men and women decreased over the 21-year period. This study clarifies the changes in age-adjusted mortality rates of HNC in Japan over time and will aid in developing targeted screening and prevention programs for HNC.

**Abstract:**

Globally, the numbers of head and neck cancer (HNC) cases and related deaths have recently increased. In Japan, few studies have examined crude or age-adjusted HNC mortality rates. Therefore, this study aimed to determine the trends in crude and age-adjusted mortality rates for HNC per million individuals in Japan from 1999 to 2019. Data on HNC-associated deaths were extracted from the national death certificate database using the *International Classification of Diseases, Tenth Revision* (*n* = 156,742). HNC mortality trends were analysed using joinpoint regression models to estimate annual percentage change (APC) and average APC (AAPC). Among men, no significant change was observed in the age-adjusted death rate trend from 1999 to 2014; however, a marked decrease was observed from 2014 to 2019. No changing point was observed in women. Age-adjusted mortality rates continuously decreased over the 21-year period, with an AAPC of −0.7% in men and −0.6% in women. In conclusion, the overall trend in age-adjusted rates of HNC-associated deaths decreased, particularly among men, in the past 5 years. These results will contribute to the formulation of medical policies to develop targeted screening and prevention programmes for HNC in Japan and determine the direction of treatment strategies.

## 1. Introduction

Head and neck cancer (HNC) is the sixth-most-common type of malignancy worldwide and comprises a diverse range of pathological origins along the upper aerodigestive tract [1,2]. HNC causes functional breathing, eating, speaking, tasting, and hearing impairments, leading to a significant decline in the quality of life. Globally, 4.1 million prevalent cases of HNC were reported in 2016 [3], and 1 million new cases were reported in 2020, with approximately 5 million related deaths [1,2]. Moreover, HNC is estimated to result in more than 700,000 annual deaths and economic losses of up to $535 billion by 2030 [4]. The incidence of HNC remains relatively low (approximately 5% of all cancers); nonetheless, the numbers of cases and related deaths have recently been increasing [1,2,3]. In Japan, the incidence of oral cancer, which accounts for the majority of HNC, is increasing [5]. Furthermore, the incidence of HNC is estimated to continue to increase in both men and women [6]. Therefore, intensive countermeasures are necessary to reduce the disease burden.

Considerable advances have been made in the treatment of HNC over the past decades. Currently available treatment methods for HNC include surgery, radiotherapy, chemotherapy, molecularly targeted therapy, and immunotherapy. Patients with HNC typically undergo these distinct therapies in combination [6,7]. Combinatory use of chemotherapy with a monoclonal antibody targeting the epidermal growth factor receptors or immune checkpoint inhibitors comprises the standard treatment for metastatic disease [8,9,10,11,12,13]. Despite the significant therapeutic developments over the past decades, only a few studies have examined annual trends in age-adjusted mortality rates.

Kawakita et al., reported that among HNCs in Japan, oral cavity, salivary gland, and oropharyngeal cancers showed an increasing trend in both men and women between 1993 and 2015 [14]. Although an epidemiological study has assessed the incidence of HNC by subsite in Japan, to the best of our knowledge, the changes in crude and age-adjusted mortality rates over time have not been adequately investigated. Katanoda et al., used the joinpoint method to comprehensively analyse trends in incidence and mortality by all cancer sites over time, but there have been no detailed studies on age-stratified mortality rates in HNC [5]. It is important to compare and evaluate age-adjusted mortality rates as well as stratified age-specific crude mortality rates in a composite manner to consider policy measures such as treatment strategies and screening. Cancer incidence alone is not sufficient to evaluate the effectiveness of a treatment method. Investigating the changes in cancer mortality over time using crude and age-adjusted mortality rates can help evaluate the effectiveness of treatment options and plan the allocation of resources needed for future cancer treatment and prevention. Therefore, this study aimed to clarify changes in age-adjusted HNC mortality over time in Japan.

## 2. Materials and Methods

### 2.1. Study Design

The study was a database-based epidemiological study.

### 2.2. Study Population

The study population included all HNC-related deaths in Japan during the study period, excluding those of patients of unknown sex. Data on HNCs were extracted from the following sources.

### 2.3. Data Source

This study was performed at Okayama University in 2022. Data were obtained from the vital statistics of death certificate database in Japan. Japanese death certificates are comprehensively collected and filled out by doctors and stored as electronic files. Data files containing basic patient information are anonymised and subsequently made available to the public. HNC mortality data were extracted from the 1999–2019 death certificate database, which uses the International Classification of Diseases, Tenth Revision (ICD-10) for coding underlying causes of death. This database is maintained by the Statistics Bureau of the Ministry of Internal Affairs and Communications with the participation of various ministries and agencies and is operated and managed by the National Statistics Center. HNC mortality was defined using ICD-10 codes [15], as follows: oral cavity (C00–C06), salivary glands (C07–C08), oropharynx (C09–C10), nasopharynx (C11), hypopharynx (C12–C13), malignant neoplasm of the lip, oral cavity, and pharynx (C14), and larynx (C32), according to previous studies [16,17,18,19]. As an exclusion criterion, data of deceased patients of unknown sex were excluded. Data of all patients were used to calculate the age-adjusted mortality rates per 100,000 individuals. Data of patients were stratified by age to calculate the crude mortality rates per 100,000 individuals, as follows: <25 years, 25–34 years, 35–44 years, 45–54 years, 55–64 years, 65–74 years, 75–84 years, and ≥85 years.

### 2.4. Statistical Analysis

Crude mortality rates were calculated by dividing the annual number of deaths by the number of individuals in that year. Age-adjusted mortality rates were calculated from HNC-associated death rates by age group and the individuals in that age group; these are presented as a figure per 100,000 individuals. The population of Japan in 1999 was used as the standard population. Age-adjusted mortality rate was calculated using a standard individual distribution that represents the age structure of the individuals being studied. This allowed for a more accurate comparison of mortality rates between individuals from different age groups, and it is commonly used in epidemiological research and public-health-related analyses. To estimate the trends in crude and age-adjusted mortality rates, the joinpoint regression model using Joinpoint Regression Program version 4.9.1.0 (April 2022; Statistical Research and Applications Branch, National Cancer Institute, Rockville, MA, USA) was applied [20]. Joinpoint regression analysis is a statistical method for the analysis of changes in trends over continuous segments of time. The significance of an increase or decrease within each segment was evaluated after identifying the best-fitting model. To compare the differences in mortality trends among subgroups, annual percentage changes (APCs) between trend-change points were determined with 95% confidence intervals (CIs). The average APC (AAPC) for the entire period was also estimated. The direct age-standardisation method was employed to calculate age-adjusted rates of HNC mortality using Japanese individuals in 1999 as the reference group, and the values are expressed as a figure per 100,000 individuals with 10-year age groups. Results with a *p*-value < 0.05 were considered to indicate statistical significance.

### 2.5. Ethics Approval

Data published by the Ministry of Health, Labour, and Welfare and Statistics Bureau of the Ministry of Internal Affairs and Communications, Japan, were used. This study was conducted in accordance with the principles embodied in the Declaration of Helsinki and was approved by the institutional review board of Okayama University Hospital (approval no. 2007-011). The requirement for informed consent was waived because this study was a retrospective analysis of routinely collected data. This study was reported following the Strengthening the Reporting of Observational Studies in Epidemiology reporting guidelines.

## 3. Results

### 3.1. Number of HNC-Associated Deaths

**Figure 1:**
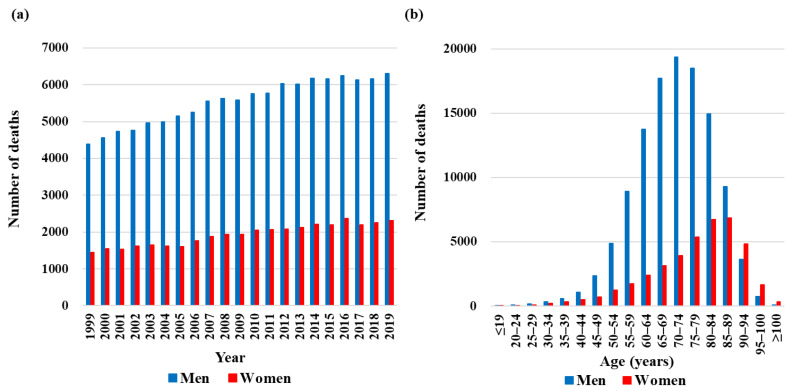
Number of head and neck cancer (HNC)-associated deaths from 1999 to 2019 in Japan. (**a**) HNC-associated deaths calculated by year and sex. (**b**) Age and sex distribution of HNC-associated deaths.

During the study period, 156,742 HNC-associated deaths (116,351 men and 40,391 women) were reported in Japan, of which 74.2% were of men. The annual number of deaths by sex is shown in Figure 1a. The total number of deaths per year increased from 5841 in 1999 to 8626 in 2019. The number of HNC-associated deaths among men increased from 4394 in 1999 to 6309 in 2019, whereas that among women increased from 1447 to 2317. The number of HNC-associated deaths by 5-year age group is shown in Figure 1b: the number of deaths peaked in the 70–74-year age group for men and in the 85–89-year age group for women.

### 3.2. Changes in HNC Mortality from 1999 to 2019

**Figure 2:**
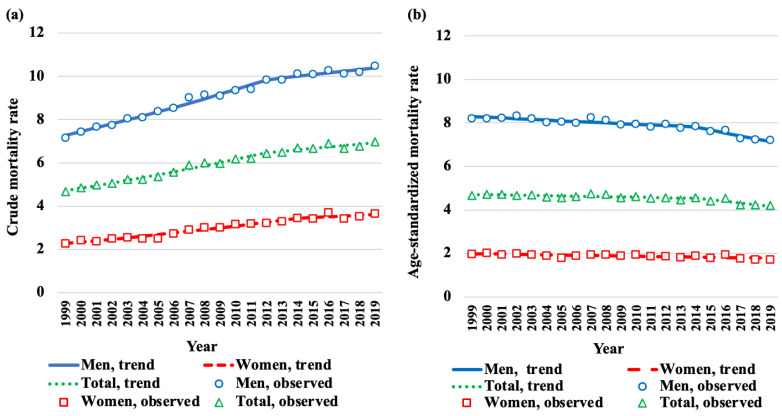
Crude and age-adjusted mortality rates of head and neck cancer (HNC) from 1999 to 2019 in Japan. (**a**) Trends in crude mortality rates of HNC, that is, deaths per 100,000 individuals, by sex. (**b**) Trends in age-adjusted rates of HNC-associated deaths per 100,000 individuals by sex.

Trends in crude mortality rates of HNC, that is, deaths per 100,000 individuals by sex, from 1999 to 2019, are shown in Figure 2a. During the study period, the overall crude mortality rate increased from 4.66 to 6.97. With respect to sex, the rate remained higher in men (7.16 to 10.48) than in women (2.26 to 3.65). By contrast, age-adjusted mortality rates showed a decrease from 4.66 to 4.20 over the 21-year period (Figure 2b). Age-adjusted mortality rates by sex decreased from 8.20 to 7.21 in men and from 1.96 to 1.71 in women. The overall trend in age-adjusted HNC mortality rates in Japan was approximately four-fold higher in men than in women. The results of the joinpoint regression analysis by sex are presented in Table 1. One inflection point was observed in 2014, both for the total study population and for men. The overall AAPC was −0.6% (95% CI: −0.9 to −0.3). No significant change was observed in APC from 1999 to 2014; however, a marked decrease in APC was observed from 2014 to 2019 (APC: −1.7%, 95% CI: −2.7 to −0.6%). Similarly, in men, APC significantly decreased during the 2014–2019 period (APC: −1.8%, 95% CI: −2.6 to −1.0%). No inflection point was observed in women. Age-adjusted mortality rates for both sexes decreased over the 21-year period, with an AAPC of −0.7% (95% CI: −1.0 to −0.5%) and −0.6% (95% CI: −0.8 to −0.3) in men and women, respectively.

**Figure 3:**
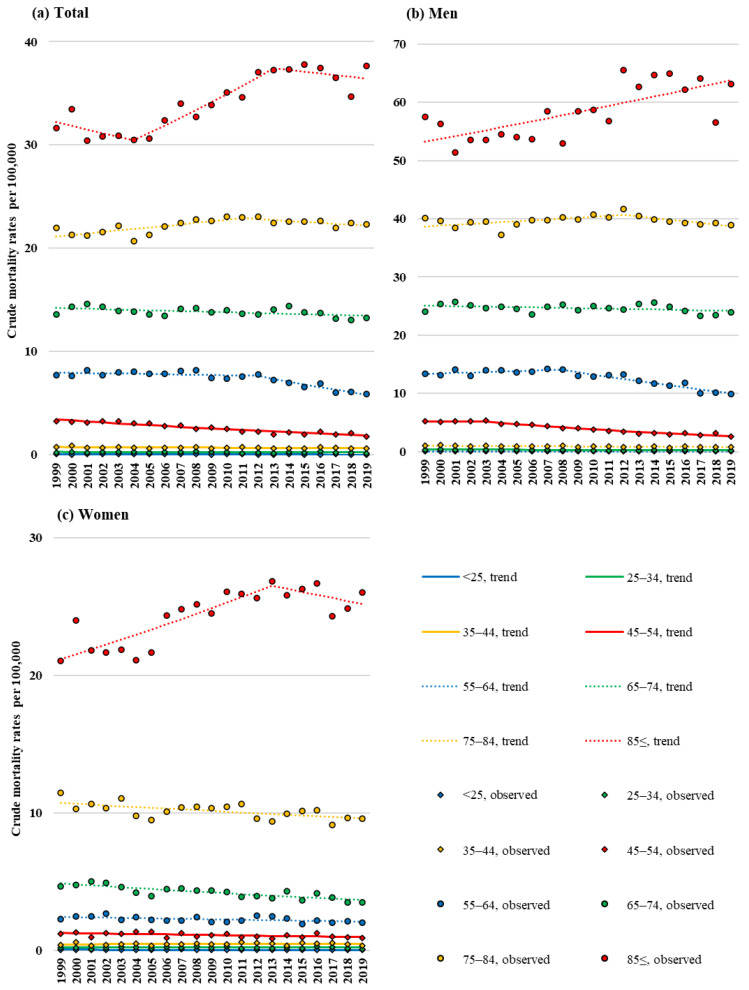
Trends in crude mortality rates of head and neck cancer (HNC), that is, deaths per 100,000 individuals, by age group and sex from 1999 to 2019: (**a**) total, (**b**) men, and (**c**) women.

Figure 3 presents the crude mortality rates of HNC categorised by 10-year age groups, including those aged ≥25 years, and sex. Table 2 summarises the results of the joinpoint regression analysis with respect to these data. Overall, the crude mortality rates decreased in the 35–44-, 45–54-, 55–64-, and 65–74-year age groups, with APCs of −0.8% (95% CI: −1.5 to −0.2%), −3.0 (95% CI: −3.4 to −2.6), −1.6% (95% CI: −2.1 to −1.0%), and −0.3% (95% CI: −0.5 to −0.1), respectively. No changes in AAPC were observed in the <25-, 25–34-, 75–84-, and ≥85-year age groups. During the study period, APC decreased in both men and women in the 45–54- and 55–64-year age groups, respectively, with APCs of −3.3% (95% CI: −4.1 to −2.6%) and −1.4% (95% CI: −2.0 to −0.9%) in men and −1.3% (95% CI: −2.2 to −0.4%) and −0.8 (95% CI: −1.3 to −0.2) in women. However, the crude mortality rates increased in both men and women in the ≥85-year age group, with AAPC values of 0.9% (95% CI: 0.5 to 1.4%) and 0.9% (95% CI: 0.2 to 1.5%), respectively. As a sensitivity analysis, age categories were classified in 10-year increments beginning at age 20, with a decreasing trend in AAPC for 50–59- and 60–69-year groups and no change in AAPC for those aged 70 and older.

## 4. Discussion

This study revealed the following major findings: In Japan, (i) the overall number of HNC-associated deaths increased 1.48-fold, and the crude mortality rate increased 1.50-fold from 1999 to 2019; (ii) age-adjusted mortality rates for HNC were four times higher in men than in women over the two decades; (iii) there has been a significant decrease in the number of HNC-associated deaths with an APC of −1.7% during the 2014–2019 period in men; and (iv) age-adjusted mortality rates in men and women decreased over time, with AAPC values of −0.7% (95% CI: −1.0 to −0.5%) and −0.6% (95% CI: −0.8 to −0.3), respectively.

In Japan, more men than women died from HNC. Siegel et al., reported that men outnumbered women in their 2022 estimates of deaths from oral cavity and pharynx cancers in the United States [21], a trend similar to that of deaths assessed by sex in Japan. Hashibe et al., reported that smoking, rather than alcohol consumption, increases the odds ratio of HNC [22]. Smoking is a representative risk factor for HNC, as observed in meta-analysis studies [23]. Smoking is a more common habit among men than among women, which may be the main reason for the high incidence of HNC among men in Japan [24]. Furthermore, women have a lower incidence of HNC, owing to female hormones, which may account for the relatively low number of deaths due to the low number of HNC cases [25]. Although the HNC-related mortality rate is rising in Japan, our study suggests that the age-adjusted mortality rate among all individuals has significantly decreased. In England, age-adjusted HNC-related mortality rates (per 100,000 individuals) from 2009 to 2011 were reported as 5.70 in men, 2.02 in women, and 3.86 overall [26]. In this study, age-adjusted mortality rates were also higher in men than in women. The age-adjusted mortality rate in men during the study period ranged from 8.20 to 7.21, which was continuously higher than that in England. For oral cavity and pharynx cancers, which are the most common HNCs and account for a large proportion of the incidence rates, age-adjusted mortality rates worldwide in 2020 were higher in men than in women [2]. Furthermore, Ferlay et al., reported a similar trend for age-adjusted mortality estimates for lip, oral cavity, and pharynx cancers in Europe for 40 countries in 2018, with men having higher rates than women [27]. Znaor et al., reported that the incidence and age-adjusted mortality rates of HNC in Croatia during the 1999–2008 study period decreased over time, and the most prominent decreases of −5.7% annually for incidence and −9.3% for mortality were observed in the youngest age group (i.e., those aged 30–39 years) [28]. In our study, crude mortality rates in the 35–44-, 45–54-, 55–64-, and 65–74-year groups decreased, with the highest decrease in the 45–54-year group. As a sensitivity analysis, age categories were classified in 10-year increments beginning at age 20, with a decreasing trend in AAPC for 50–59- and 60–69-year groups and no change in AAPC for those aged 70 and older. In men, the crude mortality rate showed a decreasing trend from the <25-year group to the 55–64-year group; however, in women, the decreasing trends were observed in the 45–54-, 55–64-, and 65–74-year groups. These trends may be due to a decrease in the incidence of smoking in Japan. In general, older adults are less tolerant of cancer chemotherapy and may not respond well to treatment. Miller et al., reported that age-adjusted mortality rates for oral and pharyngeal cancers in the United States from 2010 to 2019 increased with an AAPC of 0.4% but decreased with an AAPC of −0.8% for cancers other than those of the tongue, tonsils, and mid-pharynx [29]. Furthermore, Guo et al., reported that the 5-year survival rate for HNC in the United States increased from 54.1% to 66.8% between 1995 and 2014, a trend that may have been similar to the decline in age-adjusted mortality rates for HNC in this study [30]. Kawakita et al., reported an increase in the incidence of HNC in Japan from 1993 to 2015 [14]. The co-occurrence of an increase in the incidence of HNC and a decrease in the age-adjusted mortality rate can be partly attributed to the early detection of cancer in Japan and advances in therapeutic agents.

One potential reason for the decrease in APCs since 2014 is that, in 2012, Japan’s state insurance system started covering Cmab for HNC. Takenaka et al. reported that the mortality rate of Japanese patients with squamous cell carcinoma of the head and neck did not change between welfare and other insurance intervention groups. This fact indicated that the healthcare system in Japan is equal despite income disparity, and the relatively expensive Cmab is used at an equal frequency in the Japanese healthcare system [31]. The decrease in age-adjusted mortality rates in the 35–44-, 45–54-, 55–64-, and 65–74-year age groups may be largely related to the contribution of anticancer drug therapy, as these patients are typically eligible for cancer chemotherapy. Immunotherapy is a newly established treatment for HNC, and nivolumab has been covered by insurance since 2017 in Japan. Although the results of this study did not suggest the influence of a change in therapeutic strategy, further validation is required, owing to the short study period.

Other reasons for the decline in age-adjusted mortality rates may be the increasing rate of dental visits in Japan [24]. It has been reported that regular or frequent dental visits promote early detection of HNC and reduce the public health burden [32]. On the other hand, the dental visit rate in Japan is lower than in other developed countries, and promoting dental visits as preventive screening may be important in reducing the mortality rate of HNC in Japan [33]. Additionally, HPV is thought to be one of the causes of mid-pharyngeal cancer; HPV vaccine is available only for cervical cancer in Japan, and it may be necessary to consider improving HPV vaccination for HNC in the future [34,35,36].

This study had some limitations owing to the nature of the data and methods used. First, as the underlying causes of death were analysed from death certificates, HNC-associated death rates might have been underestimated because of underreporting. Second, owing to the absence of clinical data, comprehensive survival analyses or HNC prognosis analysis could not be performed. A third limitation is that our analysis is only descriptive. Furthermore, the cancers analysed were not grouped into clinically relevant subtypes. As mentioned above, further studies are needed to clarify the factors underlying the observed cancer trends, including analysis by clinical stage and histological type as well as modelling approaches. Finally, the accuracy of the recorded causes of death could not be determined because ICD-10 codes, C00-C14 and C32, for vital statistics were used to obtain the data. However, the quality of death certificate data in Japan is considered high compared with that in other countries [37].

## 5. Conclusions

This study revealed an increasing trend in HNC-associated crude mortality using joinpoint regression analysis. Age-adjusted mortality rates declined in both men and women, with a marked decrease in mortality rates in men after 2014. This study suggests that advances in diagnostic and therapeutic modalities for HNC may have contributed to the decline in mortality from HNC. The findings of this study indicate that there is a need to formulate medical policies, including further HNC treatment and prevention strategies for people over 75 years of age and for women who are less likely to benefit from the impact of therapeutic advances, including molecularly targeted therapies.

## Figures and Tables

**Table 1:** Trends in age-adjusted rates of head and neck cancer-associated deaths per 100,000 population by sex (1999–2019).

	Period 1		Period 2		Entire Study Period
	Years	APC (%)	Years	APC (%)	Average APC (%), (95% CI)
Total	1999–2014	−0.2	2014–2019	−1.7 ^a^	−0.6 (−0.9 to −0.3) ^a^
Men	1999–2014	−0.4	2014–2019	−1.8 ^a^	−0.7 (−1.0 to −0.5) ^a^
Women	1999–2019	−0.6 ^a^			−0.6 (−0.8 to −0.3) ^a^

^a^ Significantly different from zero (*p* < 0.05). APC, annual percentage change; CI, confidence interval.

**Table 2:** Trends in the rate of annual crude mortality for head and neck cancer per 100,000 population by sex and age group (1999–2019).

Age Group (Years)	Period 1		Period 2		Period 3		Average APC (%), (95% CI)
	Years	APC (%)	Years	APC (%)	Years	APC (%)	
Total							
<25	1999–2019	−3.0					−3.0 (−6.0 to 0.2)
25–34	1999–2019	−1.1					−1.1 (−2.7 to 0.6)
35–44	1999–2019	−0.8 ^a^					−0.8 (−1.5 to −0.2) ^a^
45–54	1999–2019	−3.0 ^a^					−3.0 (−3.4 to −2.6) ^a^
55–64	1999–2012	−0.3	2012–2019	−3.9 ^a^			−1.6 (−2.1 to −1.0) ^a^
65–74	1999–2019	−0.3 ^a^					−0.3 (−0.5 to −0.1) ^a^
75–84	1999–2011	0.7 ^a^	2011–2019	−0.4			0.2 (−0.1 to 0.5)
≤85	1999–2004	−0.8	2004–2013	2.3 ^a^	2013–2019	−0.4	0.6 (−0.2 to 1.5)
Men							
<25	1999–2019	−4.0 ^a^					−4.0 (−7.6 to −0.3) ^a^
25–34	1999–2019	−1.8 ^a^					−1.8 (−3.3 to −0.2) ^a^
35–44	1999–2019	−1.4 ^a^					−1.4 (−2.1 to −0.8) ^a^
45–54	1999–2003	−0.5	2003–2019	−4.5 ^a^			−3.3 (−4.1 to −2.6) ^a^
55–64	1999–2008	0.6	2008–2019	−3.0 ^a^			−1.4 (−2.0 to −0.9) ^a^
65–74	1999–2019	−0.2					−0.2 (−0.4 to 0.0)
75–84	1999–2012	0.4 ^a^	2012–2019	−0.7 ^a^			0.0 (−0.2 to 0.3)
≤85	1999–2019	0.9 ^a^					0.9 (0.5 to 1.4) ^a^
Women							
<25	1999–2019	−3.0					−3.0 (−8.1 to 2.3)
25–34	1999–2019	0.1					0.1 (−2.7 to 2.9)
35–44	1999–2019	0.4					0.4 (−1.0 to −1.8)
45–54	1999–2019	−1.3 ^a^					−1.3 (−2.2 to −0.4) ^a^
55–64	1999–2019	−0.8 ^a^					−0.8 (−1.3 to −0.2) ^a^
65–74	1999–2019	−1.4 ^a^					−1.4 (−1.9 to −1.0) ^a^
75–84	1999–2012	−0.6 ^a^					−0.6 (−0.9 to −0.2) ^a^
≤85	1999–2013	1.6 ^a^	2013–2019	−0.8			0.9 (0.2 to 1.5) ^a^

^a^ Significantly different from zero (*p* < 0.05). APC, annual percentage change; CI, confidence interval.

## Data Availability

The datasets generated and analysed during the current study are available from the corresponding author upon reasonable request.
